# The Temporal–Spatial Parameters of Gait After Total Knee Arthroplasty

**DOI:** 10.3390/jcm14134548

**Published:** 2025-06-26

**Authors:** Karina Szczypiór-Piasecka, Paulina Adamczewska, Łukasz Kołodziej, Paweł Ziętek

**Affiliations:** 1 Department of Rehabilitation of the Musculoskeletal System, Pomeranian Medical University, 1 Unii Lubelskiej Street, 71-252 Szczecin, Poland; lukasz.kolodziej@pum.edu.pl; 2 Student Research Group for Orthopedic Rehabilitation and Manual Therapy at the Department of Orthopedics, Traumatology and Musculoskeletal Oncology, Pomeranian Medical University, 1 Unii Lubelskiej Street, 71-252 Szczecin, Poland; 3 Autonomous Knee Surgery Unit, Pomeranian Medical University, 1 Unii Lubelskiej Street, 71-252 Szczecin, Poland; pawel.zietek@pum.edu.pl

**Keywords:** total knee arthroplasty, gait, stride length, walking speed, knee osteoarthritis

## Abstract

**Background/Objectives:** Gait abnormalities in advanced knee osteoarthritis (KOA) are characterized by decreased stride length, walking speed, and cadence. Total knee arthroplasty (TKA) is intended to improve temporal–spatial gait parameters; however, the extent and timing of functional recovery remain under investigation. To assess changes in stride length, walking speed, and cadence following TKA in short- and long-term perspectives, and to compare outcomes with a non-operated KOA cohort. **Methods:** A prospective observational study was conducted involving 46 patients with unilateral KOA (grades III–IV, Kellgren–Lawrence scale) who underwent cemented TKA via a medial parapatellar approach. Group I (*n* = 34) was assessed one day prior to surgery and six weeks postoperatively. Group II (*n* = 12), a follow-up subset, was reassessed 1.5 years postoperatively. Group III (*n* = 34) served as a non-operated control group, assessed only preoperatively. Temporal–spatial gait parameters were evaluated under standardized conditions using a two-dimensional video analysis (Kinovea^®^ software version 0.8.27). Stride length (m) and walking speed (m/s) were assessed during continuous walking along a 15 m corridor, with at least three valid gait cycles averaged per trial. Cadence (steps/min) was determined during a one-minute walk and verified frame-by-frame. No structured outpatient physiotherapy was provided; all patients followed a standardized in-hospital rehabilitation protocol. **Results:** In Group I, the mean stride length increased from 0.40 ± 0.10 m to 0.42 ± 0.10 m (*p* = 0.247), walking speed improved from 0.41 ± 0.027 m/s to 0.47 ± 0.022 m/s (*p* = 0.063), and cadence increased significantly from 72.9 ± 7.8 to 77.1 ± 8.6 steps/min (*p* = 0.044). In Group II, the mean stride length rose from 0.39 ± 0.10 m to 0.52 ± 0.09 m (*p* < 0.001), walking speed improved from 0.44 ± 0.02 m/s to 0.69 ± 0.01 m/s (*p* < 0.001), and cadence increased from 73.7 ± 8.8 to 103.6 ± 7.4 steps/min (*p* < 0.001). Compared to the control group (Group III: stride length 0.42 ± 0.09 m; walking speed 0.41 ± 0.02 m/s; cadence 73.9 ± 7.9 steps/min), Group II demonstrated superior values across all parameters (*p* < 0.001 for each comparison). No significant correlations were observed between BMI and gait outcomes. **Conclusions:** Total knee arthroplasty resulted in progressive improvement in temporal–spatial gait parameters. While early postoperative gains were limited, substantial functional restoration was observed at long-term follow-up, emphasizing the importance of extended recovery monitoring in post-TKA evaluation.

## 1. Introduction

Human gait is a complex neuromechanical process involving the synchronized activity of the central and peripheral nervous systems, the musculoskeletal structures, and sensory feedback loops. It consists of a sequence of coordinated movements that ensure stability. Central pattern generators located in the spinal cord, modulated by supraspinal signals, produce rhythmic motor patterns responsible for locomotion. Proper gait requires efficient functioning of all lower limb joints, particularly the knee joint, which acts as both a shock absorber and energy transmitter [1].

The human gait cycle is divided into two main phases: the stance phase and the swing phase. The stance phase, accounting for approximately 60% of the gait cycle, begins with the initial contact of the foot with the ground, typically the heel. During this initial contact, the knee is extended and the hip is slightly flexed, preparing the limb to bear body weight. The loading response immediately follows, during which body weight is transferred to the supporting limb. In this phase, the knee flexes to absorb shock, and stabilizing muscles activate to maintain balance.

The mid-stance phase follows, where the body moves over the stable foot. At this point, the knee extends and the hip transitions into extension. Eccentric muscle contractions control the movement of body segments over the foot. Terminal stance, the final part of the stance phase, begins as the heel lifts off the ground and body weight shifts onto the forefoot. The hip reaches maximum extension, and the knee remains slightly flexed to prepare for limb lift-off. The stance phase concludes with pre-swing, during which the toes leave the ground and the swing phase begins. During pre-swing, the knee flexes further, and the hip starts flexing again, initiating free limb movement [2,3].

The swing phase accounts for approximately 40% of the gait cycle. It begins with the initial swing, during which the lower limb lifts off the ground. The knee flexes to about 60°, and the hip deepens its flexion. In mid-swing, the limb moves forward, achieving maximum hip flexion, while the knee extends in preparation for ground contact. The cycle ends with terminal swing, when the knee reaches full extension and the muscles responsible for limb stabilization activate in preparation for the next foot–ground contact [2,3].

The energy efficiency of gait results from the use of the double-pendulum mechanism, allowing for the recovery of kinetic and potential energy, thereby minimizing energy expenditure during walking [4].

Key gait parameters include step length, stride length, cadence, walking speed, duration of gait phases (stance and swing phases), double support time, and step width. Step length is defined as the distance between the contact points of one foot with the ground and the subsequent contact of the opposite foot. In healthy adults, step length typically ranges from 0.60 to 0.80 m. Stride length, the distance between two consecutive ground contacts of the same foot, usually measures about 1.2–1.6 m [5].

Average step length depends on factors such as height, sex, and gait technique. Cadence—the number of steps taken per minute—typically ranges from 90 to 120 steps/min, depending on age, sex, and physical condition [6]. Walking speed, calculated as stride length multiplied by cadence and divided by two, averages between 1.11 and 1.66 m/s in healthy adults. Walking speed is considered a key functional indicator, strongly associated with independence and quality of life [7,8].

Maintaining balance during gait is crucial and is achieved through coordinated movements of the pelvis, hips, knees, and ankles. For example, during the stance phase, the knee flexes slightly (approximately 15°) to absorb shock and facilitate smooth movement. During the swing phase, the knee flexes up to 60°, allowing the limb to move freely [9].

Knee osteoarthritis (KOA) is a degenerative process that leads to joint deformity, structural damage, and functional impairment. It affects all joint components, including cartilage, bone, ligaments, the joint capsule, and synovial membrane [10]. The prevalence of KOA increases with age, with radiographic features observed in 19.2–27.8% of individuals aged 45 years and older [11]. KOA causes pain, joint stiffness, and muscle weakness.

The knee joint plays a central role in all phases of gait, especially in shock absorption and control of limb movement. In normal gait, the knee flexes during the swing phase and extends just before the heel strikes. In KOA patients, these patterns are disrupted, with reduced ranges of flexion and extension, increased muscle coactivation, and decreased peak joint movements, leading to inefficient, antalgic gait patterns [12].

As a result, these individuals often exhibit antalgic gait, which is characterized by a shortened stance phase on the affected limb and a relatively prolonged swing phase. This gait pattern helps minimize the duration of loading on the painful joint. This deviation from biomechanical norms results in a shortened step length and an increased number of steps. A shortened single-limb stance phase on the affected side is also typical. Incomplete knee extension decreases gait efficiency through functional limb shortening, and increased flexor strength relative to quadriceps strength is often compensated by forward trunk lean. Persistent knee flexion leads to secondary changes in the muscular system, including prolonged activation of the biceps femoris, semimembranosus, semitendinosus, and rectus femoris muscles during the stance phase. These changes significantly impact gait biomechanics.

Patients with KOA typically exhibit reduced walking speed, shorter step length, and longer double support time. Additionally, joint instability resulting from degenerative changes often leads to altered kinematics and asymmetric loading during gait [10,11,12]. Although total knee arthroplasty significantly improves joint function and alleviates pain, recent reviews have highlighted that complete restoration of physiological gait is often not achieved. Approximately 60–70% of patients regain near-normal gait patterns within the first year following surgery [4,13]. However, reduced cadence, asymmetrical stance phases, and slower walking speeds frequently persist [14,15]. These deviations may influence mobility and overall quality of life, emphasizing the need for objective gait assessment in clinical follow-up.

## 2. Aim of the Study

The aim of this study was to analyze changes in temporal–spatial gait parameters, specifically step length, cadence, and walking speed, before and after surgical treatment of knee osteoarthritis using total knee arthroplasty (TKA).

The primary objective was to assess the degree of improvement in locomotor function during the early postoperative period (six weeks) and the long-term follow-up (1.5 years) after the procedure.

Additionally, these postoperative outcomes were compared with a control group of patients with advanced knee osteoarthritis who had not undergone surgical treatment.

Given the role of the knee joint in gait biomechanics and the characteristic gait disturbances observed in advanced osteoarthritis, we hypothesized that TKA would improve the analyzed gait parameters.

## 3. Materials and Methods

This was a prospective, single-center observational study. The research was approved by the Bioethics Committee at the Pomeranian Medical University in Szczecin (No. KB-0012/81/17). The study was conducted at the Department of Orthopedics, Traumatology, and Musculoskeletal Oncology at the Pomeranian Medical University in Szczecin (Poland). It included 46 consecutive patients with knee osteoarthritis (grades III and IV according to the Kellgren–Lawrence classification) who underwent surgical treatment, including 24 women (52.2%) aged 55–84 years and 22 men (47.8%) aged 59–82 years [16]. The study was conducted over a two-year period. Participants were divided into three groups based on the timing of assessment. All surgical procedures were performed at a single center, the Department of Orthopedics, Traumatology, and Musculoskeletal Oncology at the Pomeranian Medical University in Szczecin. Patients were recruited consecutively from the surgical waiting list and qualified for inclusion according to the chronological order of scheduled surgeries. Those who met all inclusion and none of the exclusion criteria were enrolled in the corresponding study group. Randomization was not applied.

Group I included 34 patients (18 women and 16 men) who underwent functional gait assessment six weeks after undergoing total knee arthroplasty (TKA). Inclusion criteria were unilateral knee osteoarthritis (KOA) classified as grade III or IV according to the Kellgren–Lawrence scale, confirmed by clinical and radiographic assessment; no degenerative changes exceeding grade I in the contralateral knee (as assessed on anteroposterior and lateral radiographs by a radiologist); ability to ambulate independently under test conditions; provision of written informed consent for both pre- and postoperative evaluations. Exclusion criteria included musculoskeletal, neurological, or rheumatological disorders significantly affecting gait; advanced degenerative changes in the contralateral knee; previous or concurrent orthopedic procedures involving the lower limbs during the study period; withdrawal of consent; development of severe comorbidities preventing follow-up assessment; death during the observation period.

Group II was a longitudinal subset of Group I, consisting of 12 patients reassessed 1.5 years after the initial short-term postoperative assessment. These patients had previously participated in the six-week follow-up and were subsequently included in the long-term analysis. Group II was a follow-up subset of Group I and included 12 patients (6 women and 6 men) who had previously participated in Group I and agreed to take part in a follow-up assessment 1.5 years after TKA. Inclusion criteria were identical to those of Group I, with the following additional requirements: absence of postoperative complications (e.g., infection, thrombosis, implant loosening, or dislocation); no additional lower-limb orthopedic procedures between surgery and follow-up; no newly developed or ongoing comorbidities affecting gait (particularly neurological or rheumatological conditions); ability to physically attend the follow-up visit at the research site; provided renewed informed consent. Patients were excluded if they no longer met any of the above criteria at follow-up, developed or experienced progression of gait-impairing conditions, required additional orthopedic interventions, were unable to attend the follow-up visit, declined continued participation, or passed away during the study period. An additional exclusion criterion was the patient’s use of physiotherapy that was not consulted with the attending physician or physiotherapist, i.e., physiotherapy beyond the recommendations provided during hospitalization and the six-week postoperative follow-up visit.

Those who met all inclusion and none of the exclusion criteria were enrolled in the corresponding study group. Randomization was not applied.

Group III (control group) included 34 patients (15 women and 19 men) diagnosed with unilateral knee osteoarthritis (grade III or IV according to the Kellgren–Lawrence classification), who were assessed preoperatively alongside participants in Group I. At the time of inclusion, it was known that these individuals would be unable to participate in follow-up assessments due to long-distance residence, mobility limitations, or anticipated health constraints. Therefore, they were designated as a comparative control group for single-time-point preoperative analysis. All patients in Group III were recruited from the same clinical population and fulfilled identical eligibility criteria. Group III served as a baseline reference for gait parameters, without the intention of longitudinal follow-up.

Following group allocation, all patients in Groups I and II underwent postoperative rehabilitation according to a standardized in-hospital physiotherapy protocol. None of the participants in Groups I, II, or III underwent any form of prehabilitation prior to total knee arthroplasty (TKA). All patients in Groups I and II received an identical in-hospital physiotherapy program during their postoperative hospital stay. The standard rehabilitation protocol, supervised daily by a licensed physiotherapist, included range of motion (ROM) exercises for the operated knee joint, gait training with crutches, bed mobility training, and early mobilization.

Following each supervised session, patients were instructed to perform self-directed exercises several times per day, focusing on improving joint mobility and maintaining functional independence. They were also encouraged to continue ambulating with crutches according to the physiotherapy team’s recommendations.

Importantly, patients in Group II were eligible for long-term follow-up only if they had not participated in any form of outpatient or community-based physiotherapy throughout the entire postoperative period up to the 1.5-year follow-up assessment. This criterion ensured that any functional changes observed in this group reflected only the effects of surgery and standard in-hospital rehabilitation without the influence of additional physiotherapeutic interventions.

Patients in Group I also did not receive any structured outpatient physiotherapy after discharge; however, their participation in follow-up assessments was not dependent on this factor. Group III, which was assessed only preoperatively, did not undergo any postoperative rehabilitation as it was not included in the longitudinal evaluation.

To ensure the consistency and comparability of gait parameter analysis following total knee arthroplasty (TKA), the study groups were assessed for homogeneity in terms of comorbidities, physical activity levels, and the implemented rehabilitation protocol.

No significant differences were observed between the groups (Group I, Group II, and Group III) with regard to the presence of comorbid conditions. The study design ensured that participants did not present with coexisting diseases that could substantially affect gait patterns, allowing for reliable comparisons across groups.

Similarly, the level of preoperative physical activity, assessed qualitatively during the qualification process (based on patient self-report regarding daily mobility, use of assistive devices, and independence in activities of daily living), did not differ significantly between groups. All participants were independently mobile prior to surgery, and no group demonstrated a predominance of either low or high activity profiles. This consistency reduced the risk of bias due to preexisting functional disparities.

The rehabilitation protocol during hospitalization was identical for all patients after TKA (Groups I and II). It included daily supervised physiotherapy sessions comprising range of motion (ROM) exercises, gait training with crutches, early mobilization, and education on home-based exercises. After discharge, patients in Group I continued the recommended exercise routine independently at home, while patients in Group II performed only the exercises learned during their hospital stay. Importantly, none of the participants in Groups I or II received any structured outpatient physiotherapy prior to the follow-up assessment conducted 1.5 years postoperatively. Furthermore, no participant followed a different rehabilitation plan during the study period.

Given these similarities, the groups can be considered homogeneous in terms of comorbidities, preoperative physical activity level, and therapeutic intervention. This methodological consistency strengthens the validity of comparisons between pre- and postoperative gait outcomes and minimizes potential confounding factors.

**Figure 1:**
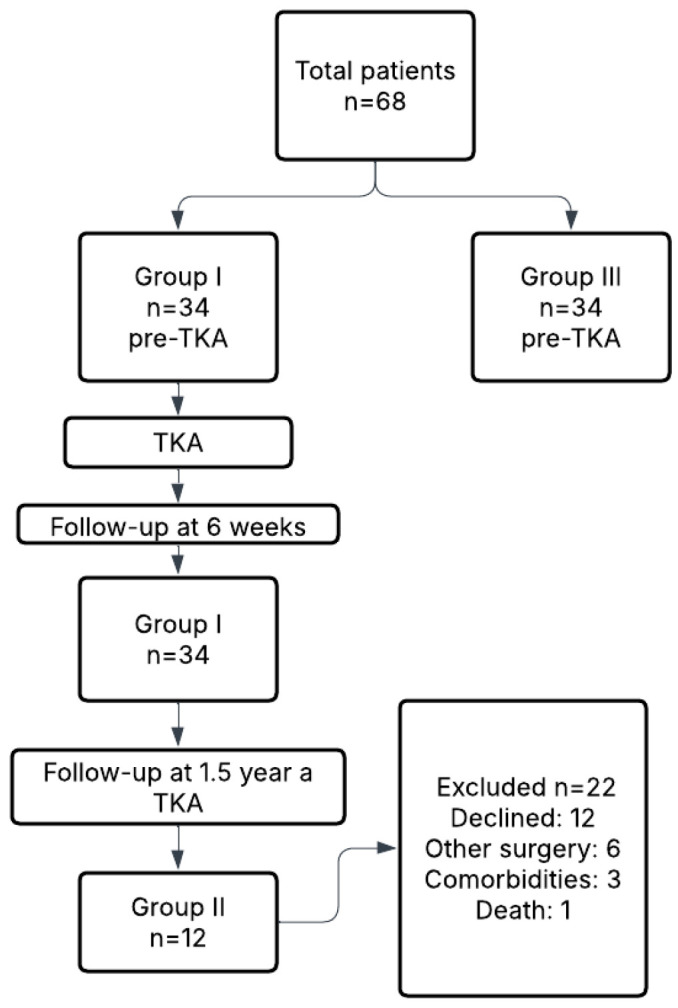
Flowchart of patient recruitment, group allocation, and follow-up.

For clarity, Group I included 34 patients (18 women, 16 men), Group II comprised a subset of 12 patients (6 women, 6 men) reassessed 1.5 years postoperatively, and Group III consisted of a separate cohort of 34 patients (15 women, 19 men) evaluated prior to TKA. All participants had no degenerative changes exceeding grade I in the contralateral knee. Radiographic assessments were performed in standing anteroposterior and lateral views and evaluated by radiology specialists. Table 1 presents the demographic characteristics of all study groups. No statistically significant differences were observed between Group I, Group II, and Group III in terms of age, body weight, height, or BMI (*p* > 0.05), confirming that the groups were demographically comparable. Specifically, Group I was used to assess short-term postoperative outcomes, Group II to evaluate long-term recovery at 1.5 years, and Group III served as a non-operated control group for baseline comparison. A flowchart illustrating participant allocation, follow-up, and reasons for exclusion from long-term analysis is presented in Figure 1.

There were no statistically significant differences in the age, gender, and BMI values between the groups.

To assess the baseline comparability of the groups regarding comorbidities and rehabilitation engagement, a chi-square test was performed across Group I (*n* = 34), Group II (*n* = 12), and Group III (*n* = 34). No statistically significant differences were found (all *p* > 0.05), confirming clinical similarity of the groups at the preoperative stage (see Table 2).

Total knee arthroplasty was performed using a cemented Vanguard^®^ knee implant (Biomet, Inc., Warsaw, IN, USA). All procedures were conducted through a medial parapatellar approach by two experienced knee arthroplasty surgeons. Subarachnoid anesthesia was administered in all patients as described by Jurewicz et al. [17,18]. The average length of hospital stay was 3.9 days (range: 3–5 days).

Among the 34 patients initially included in Group I, a subgroup of 12 patients (6 women and 6 men) met all criteria for long-term observation and was reclassified as Group II. These individuals had previously undergone standard preoperative and early postoperative assessments and were subsequently evaluated 1.5 years after TKA. No postoperative complications—such as vascular or nerve injury, infection, venous thromboembolism, prosthesis loosening, or dislocation—were observed in this subgroup.

The limited number of participants in Group II was primarily due to logistical and clinical factors. Specifically, 17 patients declined follow-up participation due to the distance between their residence and the research site. Six individuals underwent orthopedic surgery on other joints during the observation period, making them ineligible for further gait analysis. Three patients developed unrelated comorbidities that prevented them from attending follow-up, and one patient died during the study period.

### 3.1. Measurement of Temporal–Spatial Gait Parameters

The study consisted of three stages. In Stage I, participants were qualified for inclusion based on predefined criteria and assigned to one of the three study groups. In Stage II, baseline gait measurements were collected one day prior to total knee replacement (TKR). In Stage III, these measurements were repeated at six weeks postoperatively in Group I, and at 1.5 years postoperatively in Group II.

All gait tests were performed on a flat, unobstructed surface. Before each test, participants were instructed to walk naturally, simulating their typical daily gait. If the walking pattern differed noticeably from the participant’s usual locomotion (e.g., due to hesitation or irregular pacing), instructions were repeated and the trial was restarted.

Gait assessment was conducted in two parts.

(1) Stride Length and Walking Speed Assessment

Participants walked continuously for three minutes along a 15 m corridor, turning at each end to maintain continuous motion. Stride length and walking speed were measured during this task using sagittal and frontal plane video recordings. Walking speed was calculated based on the time required to cover the 15 m distance, excluding the turning phases. Stride length was defined as the distance between two consecutive contacts of the same foot (heel to heel) and was calculated across at least three consecutive steps within each trial. Each participant performed up to three trials, and the mean values of stride length and walking speed across all valid trials were used for statistical analysis.

(2) Cadence Assessment

Cadence (steps per minute) was measured during a one-minute continuous walk in a square-shaped corridor. The number of steps was recorded from video footage and manual count was performed using Kinovea’s frame-by-frame step counting function. As in the previous task, participants performed up to three trials, and the average cadence from all valid trials was calculated. Testing conditions were standardized for all participants.

A supplementary trial was conducted using a 3 m-long tape placed on the floor. After taking five preparatory steps, participants entered the marked segment, which was video recorded for additional analysis. This trial was used to qualitatively verify stride length, cadence, walking speed, and overall gait pattern. In cases of notable variability between trials, the test was repeated. Standardized verbal instructions were provided before each attempt.

All video recordings were analyzed using Kinovea^®^ software (version 0.8.27), a validated, open-source tool for two-dimensional motion analysis. Calibration was performed using a 1 m tape placed along the walking path to provide a real-world reference. Stride length (measured from heel to heel) was calculated from at least three consecutive steps during steady-state walking in each trial. Walking speed and cadence were derived from video segments using Kinovea’s integrated tools. All values were averaged across valid trials for final analysis.

Kinovea has demonstrated good agreement with three-dimensional motion capture systems such as Vicon^®^, with reported intra- and inter-rater reliability values exceeding ICC = 0.85 and measurement errors within 2 cm for linear parameters. Its accessibility and ease of use make it a reliable alternative for spatiotemporal gait analysis in clinical research settings [19,20].

### 3.2. Statistical Analysis

Statistical analyses were performed using IBM SPSS Statistics (version 26; IBM Corp., Armonk, NY, USA). The Shapiro–Wilk test was applied to assess the normality of the distribution of continuous variables. As the majority of the assessed gait parameters deviated from a normal distribution, non-parametric methods were applied in subsequent analyses.

Pre- and postoperative comparisons within the same individuals were conducted using the Wilcoxon signed-rank test. Differences between independent groups were analyzed using the Mann–Whitney U test. Correlations between gait parameters and continuous variables (e.g., BMI) were evaluated using Spearman’s rank correlation coefficient.

For comparisons involving three dependent samples, the Kendall rank correlation test was employed. A *p*-value < 0.05 was considered statistically significant. For descriptive purposes only, results with *p*-values between 0.05 and 0.1 were noted as potential tendencies, although these were not interpreted as significant findings.

All statistical analyses were performed under the supervision of a certified physiotherapist experienced in the rehabilitation of patients with knee osteoarthritis. Data analysis and interpretation were conducted in collaboration with a professional statistical consultant.

A generative AI tool (ChatGPT, OpenAI, GPT-4, 2024 version) was used solely for minor language adjustments. The scientific integrity of the manuscript was entirely maintained by the authors.

## 4. Results

### 4.1. Early Postoperative Outcomes (Group I—6 Weeks Post-TKA)

As shown in Table 3, temporal–spatial gait parameters in Group I showed partial improvement six weeks after total knee arthroplasty (TKA). Although the mean stride length increased from 0.40 m to 0.42 m (Δ = +0.02 m; range: 0.175–0.6), this change was not statistically significant (Z = –1.158; *p* = 0.247).

The walking speed rose from 0.41 m/s (range: 0.20–0.88) to 0.47 m/s (range: 0.23–1.20), yet this difference did not reach statistical significance (Z = –1.857; *p* = 0.063).

In contrast, cadence showed a statistically significant improvement, increasing from 72.9 steps/min (range: 60–92) to 77.1 steps/min (range: 64–92) (Z = –3.215; *p* = 0.044). These findings suggest that while the early postoperative period may not yield full functional restoration, improvements in gait rhythm are already observable.

To further contextualize these early postoperative changes, Table 4 compares gait parameters between Group I at 6 weeks post-TKA and the control group (Group III) with no advanced joint degeneration. The only statistically significant difference was found in walking speed, indicating that stride mechanics and cadence remained comparable at this early stage.

These early postoperative findings contrast with the more pronounced gait improvements observed in Group II at the 1.5-year follow-up, detailed in the next section.

### 4.2. Long-Term Outcomes (Group II—1.5 Years Post-TKA)

As summarized in Table 5, significant improvements were observed in all assessed gait parameters in Group II between the preoperative evaluation and the 1.5-year follow-up. The mean stride length increased from 0.39 m (range: 0.27–0.60) before surgery to 0.52 m (range: 0.42–0.68) at follow-up (*W* = 0.757; *p* < 0.001).

Similarly, walking speed rose markedly, from 0.44 m/s (range: 0.29–0.82) to 0.69 m/s (range: 0.44–1.46) (*W* = 0.674; *p* < 0.001).

The most pronounced change was observed in cadence, which increased from 73.7 steps/min (range: 60–92) to 103.6 steps/min (range: 87–121) (*W* = 0.776; *p* < 0.001). These results indicate a substantial and statistically robust recovery of gait function over the long-term postoperative period.

The effect sizes suggest clinically meaningful recovery of stride efficiency, walking speed, and rhythm.

To determine whether gait performance returned to normative levels, Table 6 compares data from Group II with Group III. Patients assessed 1.5 years after TKA outperformed controls in all gait parameters, which may reflect functional adaptation or overcompensation in response to long-term rehabilitation.

### 4.3. Between-Group Comparison of Gait Parameters

As shown in Table 7 and Table 8, patients in Group II (1.5 years post-TKA) demonstrated superior performance in all gait parameters compared to both Group I (6 weeks post-TKA) and Group III (controls). Table 7 provides mean values stratified by sex across the three study groups, while Table 8 summarizes the results of between-group statistical comparisons. Notably, significant differences were observed between Group II and both comparator groups in stride length, walking speed, and cadence. In contrast, between Group I and the control group, only walking speed differed significantly.

Group II demonstrated markedly improved stride length and walking speed compared to both Group I (U = 72; *p* = 0.0108) and the control group (U = 63; *p* = 0.0141). Although Group I showed slight improvements in gait parameters compared to the control group, only the change in walking speed reached the threshold of statistical significance (U = 9; *p* < 0.001), while differences in stride length and cadence were not significant.

Cadence peaked in Group II, with a notable improvement over both Group I (U = 216; *p* = 0.0108) and Group III (U = 189; *p* = 0.0141). No meaningful difference in cadence was observed between Group I and Group III (U = 27; *p* = 0.1613).

These cross-sectional comparisons underscore the limited short-term functional recovery at six weeks post-TKA and the substantial, time-dependent gains observed over the 1.5-year follow-up.

To further explore potential sex-related differences in postoperative gait outcomes, gait parameters were stratified by sex within each study group. As shown in Table 7, men demonstrated slightly higher stride length and walking speed values across all groups, with the greatest disparity observed in Group II (1.5 years post-TKA). Cadence values were generally higher among women, particularly in the long-term postoperative group. Although these observations are descriptive and did not reach statistical significance, they may reflect inherent sex-specific gait adaptations following TKA. Further investigation into larger, stratified cohorts is warranted to validate these preliminary findings.

Table 8 presents the results of between-group comparisons of gait parameters using the Mann–Whitney U test. As shown in Table 7, statistically significant differences in stride length, walking speed, and cadence were observed between Group II and both comparator groups. In contrast, between Group I and the control group, only walking speed differed significantly, while differences in stride length and cadence did not reach statistical significance. The analysis revealed statistically significant differences in stride length, walking speed, and cadence between Group I and Group II, as well as between Group II and the control group (Group III). In contrast, no statistically significant differences were found between Group I and the control group, except for walking speed. These findings support the conclusion that long-term postoperative improvements in gait function are more pronounced than early postoperative changes and may even exceed the normative values observed in healthy individuals.

### 4.4. Relationship Between BMI and Gait Parameters

As presented in Table 9, no statistically significant correlations were found between body mass index (BMI) and any of the evaluated gait parameters—stride length, walking speed, or cadence—in any of the study groups.

In Group I (six weeks post-TKA), the strongest observed association was a weak negative correlation between BMI and cadence (r = –0.297; *p* = 0.253), which did not reach statistical significance.

In Group II (1.5 years postoperatively), all correlation coefficients remained low (r ranging from –0.297 to 0.099), with *p*-values ranging from 0.348 to 0.861, well above the conventional threshold for statistical significance.

In Group III (control group), a weak positive correlation between BMI and stride length (r = 0.311; *p* = 0.073) approached statistical significance, suggesting a potential trend that may warrant investigation in a larger sample.

Within the scope of this study, BMI showed no clinically meaningful relationship with spatial–temporal gait parameters, either before or after TKA. These findings suggest that body composition, as measured by BMI, may play a limited role in functional gait recovery and should be interpreted with caution in the postoperative context.

### 4.5. Pain Reduction Following TKA

Subjective pain intensity, measured using the Visual Analogue Scale (VAS), showed a statistically significant reduction in both the short-term and long-term postoperative assessments (Table 10).

In Group I, VAS scores decreased from 6.97 ± 0.83 before surgery to 3.97 ± 0.83 at six weeks postoperatively (Z = –4.56; *p* < 0.001), indicating moderately early pain relief.

In Group II, the reduction was even more pronounced, with scores declining from 7.00 ± 0.85 to 0.50 ± 0.80 at 1.5 years postoperatively (Z = –3.07; *p* = 0.002), reflecting near-complete resolution of pain over time.

Between-group comparisons using the Mann–Whitney U test revealed statistically significant differences in reported pain intensity. Both postoperative groups (I and II) reported significantly lower VAS scores compared to the control group (Group III), with *p* < 0.001 in each case. Additionally, pain intensity was significantly lower in Group II compared to Group I (U = 204; *p* = 0.014).

Effect sizes for pain reduction were large in both postoperative groups (r = 0.78 in Group I and r = 0.89 in Group II), indicating a strong clinical impact of the surgical intervention.

These findings underscore the effectiveness of total knee arthroplasty not only in improving functional gait outcomes but also in providing durable and clinically meaningful pain relief, particularly in the long-term postoperative period.

### 4.6. Relationship Between Demographic Factors and Gait Parameter Changes

To explore the potential influence of demographic characteristics on gait improvements, correlation analyses were performed between changes in temporal–spatial gait parameters (stride length, walking speed, cadence) and age, BMI, and sex. No statistically significant correlations were found (all |r| < 0.2), suggesting that the improvements observed after total knee arthroplasty were independent of these demographic variables in our sample.

## 5. Discussion

The results obtained in this study do not fully align with those reported by Ogrodzka and Niedźwiedzki, who analyzed a group of 20 patients six months after total knee arthroplasty (TKA). They observed a reduction in the number of steps, stride length, and walking speed after surgery, as well as an increase in the time required to perform a single step and a longer stance phase compared to a healthy control group [21].

The average stride length observed six weeks after TKA in the present study was 0.42 m, which slightly increased from the preoperative value of 0.40 m, although this change was not statistically significant. At the long-term follow-up (1.5 years after surgery), stride length significantly improved to 0.52 m. Regarding walking speed, a slight, non-significant improvement from 0.41 m/s to 0.47 m/s was observed six weeks postoperatively, whereas a statistically significant and clinically relevant increase up to 0.69 m/s was demonstrated at 1.5 years after TKA.

The discrepancy between our early postoperative outcomes and those reported by Ogrodzka and Niedźwiedzki may be related to different follow-up durations and rehabilitation protocols. Ogrodzka and Niedźwiedzki assessed patients at six months postoperatively, whereas our early postoperative analysis was already conducted at six weeks. Longer recovery periods typically result in more pronounced gait improvements, as shown in our 1.5-year follow-up group. Abdel et al. [22] conducted a clinical trial in patients three months after TKA. The mean stride length and walking speed improved from 0.82 m and 0.64 m/s before surgery to 0.96 m and 0.76 m/s postoperatively. These outcomes represented better functional results than those observed in our study, despite Abdel’s cohort having a higher average age of 71 years. The authors suggested that extending the follow-up period to six months or one year might reveal further differences. However, Börjesson et al. demonstrated that outcomes at five years postoperatively were similar to those at three months, indicating that most improvements occur early after TKA. At 1.5 years postoperatively, improvements were noted in walking speed, stride length, and cadence.

Advanced knee osteoarthritis significantly disrupts movement patterns, and although TKA corrects pathological gait patterns, maintaining functional improvements require proper rehabilitation and convalescence.

Braito et al. examined 17 patients before and eight weeks after TKA [23]. The mean walking speed increased slightly from 0.91 m/s to 0.93 m/s, and stride length increased from 1.05 m to 1.09 m, though these changes were not statistically significant [20]. This suggests that meaningful gait normalization may depend on extended recovery timelines. Similar observations were made by Ogrodzka and Niedźwiedzki, where pathological gait persisted six months after TKA [24].

Callies et al. assessed six patients 12 months after TKA, a younger and leaner group compared to ours. They reported walking speeds of 1.29 m/s and 1.5 m/s and stride lengths of 0.67 m and 0.75 m before and after surgery, respectively, indicating improvement consistent with our findings [25].

Patterson et al. investigated whether sex, obesity, or time after surgery influenced gait quality in 43 patients (58% women). Their results indicated that men had worse gait outcomes compared to women, with men experiencing a decrease in walking speed after TKA, while women showed improvement. Unlike Patterson’s findings, improvements were observed in both sexes. However, these observations were descriptive in nature and did not reach statistical significance [26].

Urlich et al. focused exclusively on women up to 10 years post-TKA. Temporal-spatial gait parameters remained impaired even after this period, with patients walking 1.2 m/s slower than healthy controls and exhibiting shorter steps and prolonged double support times [27]. Thus, TKA does not fully restore all aspects of knee function, even long-term.

Naili et al. reported that although walking speed improved slightly one year after TKA, abnormal gait patterns persisted compared to healthy controls. Moreover, 9 out of 28 patients reported a decline in the quality of life one year after surgery, primarily those with poorer baseline outcomes [28]. In contrast, a marked improvement in walking speed was observed in our cohort 1.5 years after surgery, along with substantial improvements in subjective well-being.

Similarly, Metcalfe et al. showed that despite improved walking speed after TKA, abnormal limb loading patterns often persisted, increasing the risk of contralateral knee degeneration [29]. These findings underscore the importance of preoperative rehabilitation in optimizing postoperative gait outcomes and are consistent with our long-term observations of improved spatial gait parameters.

The persistence of gait alterations has also been described by Okita et al., who demonstrated that asymmetrical biomechanics may endure for years after surgery due to adaptive strategies aimed at protecting the reconstructed knee [30]. This aligns with our observation of residual deviations despite measurable improvements.

Fenner et al. reported that only 20% of TKA patients restored normal gait patterns postoperatively, even when walking speeds matched those of healthy controls [31]. Patients with better knee function achieved better gait outcomes, which is consistent with our results.

Bonnefoy-Mazure et al. found that although gait parameters worsened at three months postoperatively, physiological gait patterns were largely restored by one year [32]. These findings closely align with our 1.5-year results.

In a subsequent study, the same authors reported that patients with higher BMI had slower walking speeds and reduced knee range of motion both before and after TKA [33]. In the present study, no statistically significant correlations were found between BMI and the analyzed gait parameters. However, in the control group, a trend toward a positive correlation between BMI and stride length was observed (r = 0.311; *p* = 0.073), suggesting a potential relationship that should be investigated in larger samples.

Maier et al. used three-dimensional gait analysis in patients with persistent postoperative pain and reported significant reductions in walking speed and stride length [34]. In contrast, no chronic postoperative pain was observed in our cohort—particularly in Group II, evaluated 1.5 years after surgery, where VAS scores averaged 0.50 ± 0.80. This likely contributed to the superior functional gains observed in this group.

These findings reinforce the overall effectiveness of total knee arthroplasty in not only improving biomechanical gait outcomes but also delivering clinically meaningful, durable pain relief. The absence of long-term pain complaints may explain the enhanced quality of life reported by patients after surgery.

Consistent with our results, Singhi et al. found that although stride length was reduced in the operated limb, TKA improved overall gait efficiency [35]. Our data reflect a similar recovery pattern with functional gains emerging early and progressing over time.

Despite these positive findings, the present study has several limitations. Most notably, the long-term follow-up group (Group II) included only six participants, which limits generalizability and increases the risk of type II error. These patients represented a distinct cohort rather than a continuation of the short-term sample, and their reduced participation was largely due to logistical barriers and emerging comorbidities, as discussed in the Materials and Methods Section. Furthermore, the progressive nature of osteoarthritis—particularly degenerative changes in the contralateral knee, already evident preoperatively—could have influenced long-term gait outcomes. Finally, variability in the course and quality of postoperative rehabilitation, including access to physical therapy, adherence to home programs, and outpatient services, was not standardized or controlled, potentially introducing bias into functional recovery results [19].

Our findings indicate that demographic factors such as age, BMI, and sex did not significantly influence the extent of gait improvements following total knee arthroplasty. This is consistent with previous studies suggesting that functional recovery in terms of gait may be largely independent of these patient characteristics. However, it is important to acknowledge that the relatively small sample size limits the generalizability of these results. Future research with larger cohorts is needed to further explore potential demographic influences on postoperative gait rehabilitation.

Another important limitation is the lack of control for patient lifestyle factors, such as physical activity levels, comorbidities, and preoperative fitness, which may affect postoperative gait adaptations independently of surgical outcomes. These individual differences could contribute to both inter- and intra-group variability in gait parameter progression. The absence of a healthy normative group should be acknowledged as a limitation, as it restricts the biomechanical interpretation of the results.

Additionally, the progression of degenerative changes in the contralateral limb was not systematically monitored throughout the study period. Although patients with advanced bilateral knee OA were excluded at baseline, subclinical or progressive changes in the non-operated knee may have influenced gait symmetry and performance over time [29]. This factor is particularly relevant for long-term assessments, where compensatory gait patterns may develop in response to new or worsening pathology on the contralateral side.

Finally, we did not include advanced diagnostic tools such as acoustic gait analysis or MRI-based grading of cartilage degeneration. These methods have shown potential in identifying early biomechanical abnormalities and joint deterioration not visible on plain radiographs. For instance, machine learning models using acoustic signals have demonstrated promise in the non-invasive detection of OA-related gait patterns [36]. Moreover, MRI often underestimates the severity of cartilage lesions, which could lead to misclassification of joint status and impact the interpretation of gait outcomes [37].

Future research should aim to include larger, stratified samples, longitudinal imaging of both knees, and consistent rehabilitation protocols to minimize confounding factors and better elucidate the functional trajectory after TKA.

Although this study utilized standard temporal–spatial gait parameters assessed through observational methods and video recordings, the choice of methodology was primarily dictated by the clinical setting and the aim to ensure simplicity and repeatability of measurements in routine postoperative care. The tools applied enabled the collection of reliable data while minimizing patient burden during the perioperative period.

Nevertheless, future research could benefit from the inclusion of more advanced technologies such as three-dimensional motion capture systems and inertial measurement units (IMUs). These methods provide precise kinematic and kinetic data and allow for the detection of subtle asymmetries or compensatory strategies that may not be observable through conventional analysis.

An additional advantage of IMU-based systems is their portability and the potential for long-term gait monitoring in real-life settings, thereby improving ecological validity and sensitivity of assessments. The integration of such technologies with traditional clinical evaluation may, in future studies, contribute to a deeper understanding of adaptive mechanisms following total knee arthroplasty and support the development of more individualized rehabilitation protocols.

### Post Hoc Power Analysis

Due to the limited sample size in Group II (*n* = 12), a post hoc power analysis was conducted to assess the statistical strength of the observed changes in gait parameters over the 1.5-year postoperative period. Effect sizes (Cohen’s d) were calculated for three primary outcomes:

Stride length (pre: M = 0.39, SD = 0.099; post: M = 0.52, SD = 0.090): d = 0.97.
Walking speed (pre: M = 0.44, SD = 0.022; post: M = 0.69, SD = 0.014): d = 9.59.
Cadence (pre: M = 73.7, SD = 8.75; post: M = 103.6, SD = 7.44): d = 2.60.

Using a significance level of α = 0.05 and a two-tailed paired-samples *t*-test framework, the calculated statistical power for all three variables exceeded 0.99. This indicates a very high probability of detecting true effects despite the small sample size.

Notably, the effect size for walking speed was extremely large. This may partially reflect the very small variability in the data (low SD), which can inflate d values. Caution is therefore advised when interpreting this result.

Overall, these findings suggest that the magnitude of change in gait performance following TKA was sufficiently substantial to yield statistically robust outcomes, even in a limited cohort.

## 6. Conclusions

1. Total knee arthroplasty (TKA) contributes to significant improvements in temporal–spatial gait parameters, particularly stride length, walking speed, and cadence. These improvements become more pronounced over time, with the most substantial gains observed 1.5 years after surgery.
2. In the early postoperative period (six weeks), changes in gait parameters are present but not always statistically significant. This may be attributed to the limited convalescence period and the early phase of rehabilitation. However, a statistically significant improvement in cadence was already evident at this stage.
3. Long-term follow-up (1.5 years after TKA) reveals statistically significant increases in all analyzed gait parameters, confirming that time plays a crucial role in achieving full functional recovery.

## Figures and Tables

**Table 1:** Statistical features describing the study groups.

	Group I *n* = 34			Group II *n* = 12			Group III (Control) *n* = 34		
Number	Total *n* = 34	Women *n* = 18	Men *n* = 16	Total *n* = 12	Women *n* = 6	Men *n* = 6	Total *n* = 34	Women *n* = 15	Men *n* = 19
Parameter	X (SD)	X (SD)	X (SD)	X (SD)	X (SD)	X (SD)	X (SD)	X (SD)	X (SD)
Age (years)	71 (7.12) Range: 55–84	70.5 (7.26) Range: 55–84	71.9 (6.98) Range: 59–80	68.33 (6.43) Range: 56–82	69 (5.87) Range: 56–78	68.67 (7.53) Range: 59–82	69.88 (6.53) Range: 57.6–84	71.14 (5.22) Range: 63.9–81.7	69.42 (6.98) Range: 57.6–84
Body weight (kg)	79.2 (13.34)	74.2 (10.00)	89.5 (13.69)	83.2 (13.71)	74 (7.92)	92.3 (12.21)	79.67 (14.01)	75.02 (12.19)	81.34 (14.46)
Height (m)	1.65 (0.086)	1.61 (0.051)	1.75 (0.067)	1.67 (0.095)	1.59 (0.04)	1.75 (0.068)	1.64 (0.05)	1.63 (0.05)	1.65 (0.05)
BMI (kg/m^2^)	28.87 (3.59) Range: 21.2–36.3	28.65 (3.39) Range: 22.3–34.4	29.33 (4.05) Range: 21.2–36.3	29.7 (3.75) Range: 21.5–34.2	29.1 (3.48) Range: 22.8–34.2	30.3 (4.25) Range: 21.9–34.1	29.04 (2.80) Range: 24.6–34.8	29.3 (3.06) Range: 24.6–33.8	28.9 (2.76) Range: 24.8–34.8

Abbreviations: *n*—number of patients; X—mean; SD—standard deviation; range: minimum–maximum values.

**Table 2:** Comparison of baseline clinical characteristics across study groups.

Variable	Group I *n* = 34 (%)	Group II *n* = 12 (%)	Group III *n* = 34 (%)	*p*-Value
Hypertension	11 (32.4%)	3 (25%)	16 (47.1%)	0.285
Diabetes	8 (23.5%)	4 (33.3%)	7 (20.6%)	0.671
Obesity	13 (38.2%)	7 (58.3%)	8 (23.5)	0.082
Cardiovascular Disease	9 (26.5%)	5 (41.7%)	6 (17.6%)	0.247
Outpatient Physio	7 (20.6%)	2 (16.7%)	12 (35.3)	0.277

**Table 3:** Temporal–spatial gait parameters before and 6 weeks after total knee arthroplasty in Group I (*n* = 34).

Group I	Preoperative, *n* = 34		Postoperative (6 Weeks), *n* = 34		The Wilcoxon Test	
	Range	X (SD)	Range	X (SD)	Z	*p*
Stride length (m)	0.175–0.6	0.4 (0.097)	0.21–0.75	0.42 (0.102)	−1.158	0.247
Walking speed (m/s)	0.2–0.88	0.41 (0.027)	0.23–1.2	0.47 (0.022)	−1.857	0.063
Cadence (steps per minute)	60–92	72,9 (7.75)	64–92	77.06 (8.59)	−3.215	0.044

Abbreviations: *n*—number of patients; X—mean; SD—standard deviation; range: minimum–maximum values. Effect size (r) for cadence improvement: 0.55, indicating a large effect. Effect sizes for stride length and walking speed were 0.20 and 0.32, respectively, reflecting small-to-moderate effects.

**Table 4:** Comparison of gait parameters between patients 6 weeks after total knee arthroplasty (Group I) and the control group (Group III).

	Group I Postoperative (6 Weeks) *n* = 34		Group III (Control) *n* = 34		Mann–Whitney U Test
	Range	X (SD)	Range	X (SD)	*p*
Stride length (m)	0.21–0.75	0.42 (0.102)	0.26–0.60	0.42 (0.092)	0.272
Walking speed (m/s)	0.23–1.2	0.47 (0.022)	0.37–0.48	0.41 (0.024)	<0.001
Cadence (steps per minute)	64–92	77.06 (8.59)	60–91.4	73.9 (7.9)	0.171

Abbreviations: *n*—number of patients; X—mean; SD—standard deviation; range: minimum–maximum values. Effect size (r) for walking speed difference: 0.87, indicating a large effect. No meaningful effect observed for stride length or cadence.

**Table 5:** Preoperative and 1.5-year postoperative gait parameters in Group II (*n* = 12).

Group II	Preoperative, *n* = 12		Postoperative (1.5 Years), *n* = 12		Kendall’s W	
	Range	X (SD)	Range	X (SD)	W	*p*
Stride length (m)	0.27–0.60	0.39 (0.099)	0.42–0.68	0.52 (0.090)	0.757	<0.001
Walking speed (m/s)	0.29–0.82	0.44 (0.022)	0.44–1.46	0.69 (0.014)	0.674	<0.001
Cadence (steps per minute)	60–92	73.7 (8.75)	87–121	103.6 (7.44)	0.776	<0.001

Abbreviations: *n*—number of patients; X—mean; SD—standard deviation; range: minimum–maximum values. Effect sizes (Kendall’s W) for stride length, walking speed, and cadence were 0.757, 0.674, and 0.776, respectively, indicating strong agreement and large effect magnitudes.

**Table 6:** Comparison of gait parameters between Group II (1.5 years post-TKA) and the control group (Group III).

	Group II Postoperative (1.5 Years) *n* = 12		Group III (Control) *n* = 34		Mann–Whitney U Test
	Range	X (SD)	Range	X (SD)	*p*
Stride length (m)	0.42–0.68	0.52 (0.090)	0.26–0.60	0.42 (0.092)	0.00053
Walking speed (m/s)	0.44–1.46	0.69 (0.014)	0.37–0.48	0.41 (0.024)	<0.001
Cadence (steps per minute)	87–121	103.6 (7.44)	60–91.4	73.9 (7.9)	<0.001

Abbreviations: *n*—number of patients; X—mean; SD—standard deviation; range: minimum–maximum values. Between-group differences yielded large effect sizes: r = 0.68 for stride length, r = 0.85 for walking speed, and r = 0.79 for cadence.

**Table 7:** Cross-sectional comparison of gait parameters in Groups I, II, and III, stratified by sex.

	Group I *n* = 34			Group II *n* = 12			Group III (Control) *n* = 34		
Group	Total *n* = 34	Women *n* = 18	Men *n* = 16	Total *n* = 12	Women *n* = 6	Men *n* = 6	Total *n* = 34	Women *n* = 15	Men *n* = 19
Parameter	X (SD)	X (SD)	X (SD)	X (SD)	X (SD)	X (SD)	X (SD)	X (SD)	X (SD)
Stride length (m)	0.42 (0.1)	0.4 (0.06)	0.43 (0.06)	0.52 (0.09)	0.5 (0.06)	0.54 (0.05)	0.42 (0.09)	0.4 (0.05)	0.44 (0.05)
Walking speed (m/s)	0.47 (0.02)	0.45 (0.03)	0.49 (0.03)	0.69 (0.01)	0.66 (0.04)	0.72 (0.05)	0.41 (0.02)	0.39 (0.03)	0.43 (0.03)
Cadence (steps per minute)	77.1 (8.6)	78.3 (5.0)	75.5 (5.5)	103.6 (7.4)	107.2 (5.0)	100 (6.0)	73.9 (7.9)	76.2 (5.0)	71.9 (5.5)

Abbreviations: *n*—number of patients; X—mean; SD—standard deviation.

**Table 8:** Comparison of gait parameters between study groups based on Mann–Whitney U test.

	Group I *n* = 34		Group II *n* = 12		Group III (Control) *n* = 34	
Group	Mann–Whitney U	*p*	Mann–Whitney U	*p*	Mann–Whitney U	*p*
Stride length (m)	72	0.0108	9	<0.001	63	0.0141
Walking speed (m/s)	72	0.0108	9	<0.001	63	0.0141
Cadence (steps per minute)	216	0.0108	27	<0.001	189	0.0141

Abbreviations: *n*—number of patients. All statistically significant comparisons yielded large effect sizes (r > 0.6), supporting the robustness of Group II improvements over Groups I and III. No meaningful effects were observed between Groups I and III in stride length or cadence.

**Table 9:** Spearman’s rank correlation coefficients (r) and corresponding *p*-values between BMI and gait parameters in Groups I, II, and III.

BMI	Group I *n* = 34		Group II *n* = 12		Group III *n* = 34	
Parameter	Spearman R	*p*	Spearman R	*p*	Spearman R	*p*
Stride length (m)	−0.036	0.84	−0.297	0.348	0.311	0.073
Walking speed (m/s)	0.049	0.785	−0.057	0.861	0.057	0.746
Cadence (steps per minute)	−0.297	0.253	0.099	0.759	−0.016	0.366

All observed correlations were weak (|r| < 0.3) and statistically non-significant.

**Table 10:** Subjective pain intensity (VAS) before and after total knee arthroplasty: within- and between-group comparisons.

Group	*n*	Preoperative VAS X (SD)	Follow-Up VAS X (SD)	Within-Group Comparison (Z/*p*)	Compared to Control (Group III) (U/*p*)	Compared to Group I (U/*p*)
I	34	6.97 (0.83)	3.97 (0.83)	Z = –4.56; *p* < 0.001	U = 302; *p* < 0.001	—
II	12	7.0 (0.85)	0.50 (0.80)	Z = –3.07; *p* = 0.002	U = 18; *p* < 0.001	U = 204; *p* = 0.014
III	34	6.85 (0.81)	—		—	—

Abbreviations: *n*—number of patients; X—mean; SD—standard deviation.

## Data Availability

The data supporting the findings of this study are not publicly available due to privacy or ethical restrictions.
